# External validation of an artificial intelligence–based ECG algorithm for the early diagnosis of acute myocardial infarction: a prospective diagnostic cohort study

**DOI:** 10.1016/j.lanepe.2026.101912

**Published:** 2026-11-07

**Authors:** Arnaud Champetier, Min Sung Lee, Pedro Lopez-Ayala, Yong-Yeon Jo, Jasper Boeddinghaus, Luca Koechlin, Paolo Bima, Luca Crisanti, Tobias Zimmermann, Kyuseok Kim, Kyung Su Kim, Sunyoung Yoon, Emel Kaplan, Danielle M. Gualandro, Lourdes Herraiz Recuenco, Gabrielle Huré, Tzu-Ying Tsao, Marco Nani, Hak Seung Lee, Joon-myoung Kwon, Michael Christ, Niclas Geldermann, Òscar Miró, Ksenija Slankamenac, Roland Bingisser, Christian Nickel, Philippe Cattin, Felix Mahfoud, Ivo Strebel, Christian Mueller, APACE and APACE-Light investigators, Karin Wildi, Markku Mund, Carlos Spagnuolo, Jonas Glaeser, Thomas Nestelberger, Desiree Wussler, Maria Rubini Gimenez, Jude Formambuh, Christian Puelacher, Jeanne du Fay de Lavallaz, Julia Reinhardt, Kathrin Meissner, Katharina Rentsch, Dagmar Keller, Beatriz López, Gemma Martinez-Nadal, Esther Rodriguez Adrada, Arnold von Eckardstein, Damian Kawecki, Piotr Muzyk, Nicolas Geigy, Eliška Potluková, Stephan Steuer, Angelika Hammerer-Lercher, Andreas Buser

**Affiliations:** a Cardiovascular Research Institute Basel (CRIB) and Department of Cardiology, University Hospital Basel, University of Basel, Basel, Switzerland; b Digital Healthcare Institute, Sejong Medical Research Institute, Bucheon, Republic of Korea; c Medical AI Co., Ltd., Seoul, Republic of Korea; d Department of Cardiac Surgery, University Hospital Zurich, University of Zurich, Zurich, Switzerland; e Intensive Care Unit, Department of Acute Medicine, University Hospital Basel, University of Basel, Basel, Switzerland; f Department of Emergency Medicine, CHA Bundang Medical Center, CHA University School of Medicine, Bundang, Seongnam, Republic of Korea; g Emergency Department, Kantonsspital Luzern, Lucerne, Switzerland; h Emergency Department, Hospital Clinic, Barcelona, Catalonia, Spain; i Emergency Department, University Hospital Zurich, University of Zurich, Zurich, Switzerland; j Emergency Department, University Hospital Basel, University of Basel, Basel, Switzerland; k Department of Biomedical Engineering, University of Basel, Allschwil, Switzerland

**Keywords:** Machine learning, Myocardial infarction, Electrocardiogram, AI-ECG, External validation, Troponin

## Abstract

**Background:**

A novel artificial intelligence–based electrocardiogram (AI-ECG) model (AiTiA MI v2.00.00) for the early diagnosis of acute myocardial infarction (AMI) was developed using data from Korean patients. Its performance in a European population remains unknown.

**Methods:**

Patients presenting to the emergency department (ED) with suspected AMI were enrolled in two prospective European cohorts (APACE & APACE-Light; ClinicalTrials.gov number, NCT00470587). The primary outcome was AMI, centrally adjudicated according to the fourth Universal Definition of MI. The AI-ECG model was applied to 12-lead ECGs recorded at ED presentation. Its diagnostic performance for AMI was directly compared to that of the BASEL score, high-sensitivity cardiac troponin T (hs-cTnT), the recalibrated HEART score (all at ED presentation), and integrated physician judgment at 90 min, with the last two only available in APACE. Predefined AI-ECG triage thresholds for early rule-out and rule-in of AMI were also evaluated.

**Findings:**

Among 12,630 patients with an available digital 12-lead ECG (5047 in APACE and 7583 in APACE-Light), adjudicated AMI was diagnosed in 19% (968/5047) and 18% (1386/7583) of patients, respectively. In patients with hs-cTnT at presentation (n = 5031 and n = 7565), the AI-ECG model showed good diagnostic performance for AMI with an area under the receiver operating characteristic curve (AUC) of 0.78 (95% CI: 0.77–0.80) and 0.84 (0.83–0.85) and an area under the precision–recall curve (AUPRC) of 0.56 (0.53–0.59) and 0.65 (0.62–0.67). Its discrimination was inferior to hs-cTnT (AUC 0.94 [0.93–0.95] and 0.93 [0.92–0.94]). The AI-ECG model provided some incremental diagnostic value when combined with hs-cTnT (AUC 0.95 [0.94–0.96] and 0.95 [0.94–0.95], p < 0.001). The predefined triage thresholds ruled out 14% (706/5031) and 19% (1437/7565) of patients, with a sensitivity for AMI of 97.6% (96.5–98.5) and 97.5% (96.6–98.3), respectively.

**Interpretation:**

AI-ECG showed high, but not very high, diagnostic accuracy for AMI. As a diagnosis of AMI is unlikely to be ruled out on ECG alone, combining AI-ECG with hs-cTnT may improve decision making in the ED, warranting prospective studies.

**Funding:**

The Swiss National Science Foundation, the Swiss Heart Foundation, Innosuisse, the University Hospital Basel, the University of Basel, Abbott, Beckman Coulter, Idorsia, LSI Medience Corporation, Roche, Ortho Clinical Diagnostics, Quidel, Siemens, SpinChip, Upstream, and Singulex, the Korea Health Industry Development Institute (KHIDI), funded by the Ministry of Health & Welfare, Republic of Korea (grant number: RS-2026-25543703).

**Research in context**

**Evidence before this study**

Early and accurate identification of acute myocardial infarction (AMI) among patients presenting with non-traumatic chest pain to the emergency department is critical for timely intervention and improved outcomes. Current guidelines recommend a two-step diagnostic approach: assessment of the 12-lead electrocardiogram (ECG) followed by measurement of high-sensitivity cardiac troponin (hs-cTn). However, ECG interpretation depends on individual clinical expertise, follows rigid diagnostic criteria, and does not provide a continuous probabilistic output. We systematically searched PubMed for studies published from May 2025 to February 2026, using the keywords: “AiTiA MI”, “ROMIAE”, and “validation” without language restrictions. One study was identified, reporting external validation of the AiTiA MI algorithm using the ROMIAE study. The study describes an artificial intelligence-based ECG model, AiTiA MI v2.00.00, which was developed to predict AMI in Korean populations and demonstrated strong diagnostic performance in geographically similar cohorts. However, its performance and incremental diagnostic value as an adjunct to hs-cTn in large European cohorts have yet to be established.

**Added value of this study**

To our knowledge, this study represents the first external validation of the AiTiA MI v2.00.00 model in two large, prospective European cohorts, evaluating its diagnostic accuracy, discrimination, and incremental value over hs-cTnT in patients presenting with chest pain to the emergency department.

**Implications of all the available evidence**

While inferior to hs-cTnT at ED presentation and established clinical assessment, AiTiA MI v2.00.00 offers incremental value when combined with hs-cTn, and may serve as a useful adjunct rather than a standalone decision-making tool.

## Introduction

Acute myocardial infarction (AMI) remains a leading cause of death worldwide, yet outcomes improve substantially with early reperfusion therapy.^1^ Rapid and accurate risk stratification of patients presenting to the emergency department (ED) with non-traumatic chest pain is essential to timely guide management.^2^, ^3^ The 12-lead electrocardiogram (ECG) is the first-line tool and is pivotal for identifying patients requiring immediate invasive intervention.^4^ Ischaemic ECG changes consistent with ST-segment elevation myocardial infarction (STEMI) mandate urgent reperfusion, most commonly via primary percutaneous coronary intervention.^4^ Complementary to ECG findings, current guidelines recommend serial measurement of high-sensitivity cardiac troponin (hs-cTn) to rule-in or rule out AMI with high diagnostic accuracy.^2^, ^3^

However, the rapid identification of patients with AMI still has notable limitations. First, ECG interpretation depends on individual expertise.^5^ Second, ECG analysis follows rigid rule-based criteria and lacks continuous probabilistic estimation.^6^ Third, turnaround time of cardiac troponin often exceeds 1 h when using a central laboratory assay, delaying immediate rule-in. Fourth, the optimal combination of 12-lead ECG findings and hs-cTn concentrations remains uncertain.

Recent advances in machine learning, particularly deep learning (DL), have demonstrated strong promise for cardiovascular diagnostics based on ECG data alone.^7^, ^8^, ^9^, ^10^, ^11^, ^12^, ^13^, ^14^ The AiTiA MI v2.00.00 algorithm, an artificial intelligence–based ECG (AI-ECG) model, along with its triage thresholds, was developed in Korean populations and subsequently validated in Korean EDs.^15^ However, external validation in large, prospective, multicentre, geographically distinct cohorts is lacking. Consequently, the robustness and generalisability of its diagnostic discrimination and the proposed AI-ECG model triage thresholds remain uncertain.

Therefore, we aimed to (1) assess the diagnostic performance of the AiTiA MI v2.00.00 algorithm in direct comparison with established risk scores, hs-cTn, and integrated physician judgment (including the 12-lead ECG and the first hs-cTn measurement); (2) assess the possible incremental value of AI-ECG to hs-cTn; and (3) evaluate the diagnostic and prognostic performance of predefined AI-ECG triage thresholds within an ED pathway in two large prospective European cohorts with adjudication of AMI.

## Methods

### Study design and population

In this secondary analysis of two prospective diagnostic cohorts, adult patients presenting with non-traumatic acute chest discomfort possibly indicating AMI were enrolled in the APACE study from 04/2006 to 09/2020 (Advantageous Predictors of Acute Coronary Syndromes Evaluation, www.clinicaltrials.gov; NCT00470587), an international multicentre study, and in APACE-Light, a single-centre study conducted in Basel, Switzerland from 01/2019 to 01/2021.^16^, ^17^, ^18^, ^19^, ^20^, ^21^ APACE-Light is a prospective observational registry in which eligible patients were identified through weekly screening according to predefined criteria. Prespecified variables were extracted from routine clinical records and cleaned, and the final diagnosis was centrally adjudicated. As the registry involved no additional patient contact or study-specific procedures, it was not registered as a clinical trial.^21^

Patients were eligible for this analysis if they had a digital 12-lead ECG obtained at ED presentation. Patients were excluded if their final diagnosis remained unclear even after central adjudication, if they had end-stage renal disease on chronic dialysis, or if the AI-ECG was unable to output a valid prediction. Patients without hs-cTnT at ED presentation were excluded for the direct comparison with AiTiA MI v2.00.00 algorithm. Sex (female or male) was recorded as documented in the medical records. Ethnicity and disability status were not collected.

### Ethics

The studies were carried out according to the principles of the Declaration of Helsinki and approved by each local ethics committee and centrally approved in Basel by the Ethikkommission Nordwest-und Zentralschweiz (EKNZ) (EKNZ-nr. for APACE: 280/05, EKNZ-nr. for APACE-Light: 2020-01124). APACE required written informed consent for participation, whereas APACE-Light enrolled all consecutive eligible patients with waived informed consent, as it evaluated an established standard-of-care algorithm using only routinely collected hospital data and the national death registry, without patient contact. Reporting follows the TRIPOD+AI (Transparent Reporting of a Multivariable Prediction Model for Individual Prognosis or Diagnosis—Artificial Intelligence) statement (Supplemental Material).^22^

### Outcomes

The primary outcome was AMI, defined according to current guidelines and the 4th Universal Definition of Myocardial Infarction (UDMI).^16^, ^17^, ^23^ In APACE and APACE-Light, the final diagnosis of AMI, including its subclassification, was centrally adjudicated. All available medical records, including cardiac imaging and local serial cTn, were reviewed alongside hs-cTnT gen5 levels, which were centrally measured in all patients throughout the study period. The diagnostic procedures, routine clinical assessment, and adjudication process have been described in detail previously.^16^, ^17^ The secondary outcome was the composite of AMI (excluding index event) or all-cause death within 5 years (available in APACE).

### AiTiA MI v2.00.00: an AI-ECG model for AMI

An AI–ECG model, referred to as AiTiA MI version 2.00.00 (Medical AI Co., Ltd., Seoul, Republic of Korea), is a deep learning algorithm that estimates the AMI probability from raw 12-lead ECG recordings. This version builds upon a self-supervised ECG foundation model finetuned with the foundational datasets of AiTiA MI version 1.00.00 and the extensive clinical data from 14 EDs in the ROMIAE study cohort.^24^ In the present study, AiTiA MI version 2.00.00 was applied as a fixed, pre-trained algorithm accessed via an application programming interface (API), without retraining or updating on our cohort data. Further specifics on the development and technical validation of the algorithm are available in the Supplement.

### ECG collection and preprocessing

In the APACE and APACE-Light cohorts, digital 12-lead ECGs were recorded using different Schiller or Philips hardware. Each ECG was of 10-s duration and recorded with a sampling rate of 500 Hz or 1000 Hz. ECGs recorded at a sampling rate of 1000 Hz were downsampled to 500 Hz by discarding every second sample. For each patient, only the first ECG performed at the ED was selected and all of them were recorded before possible coronary angiography.

### Direct comparison: risk scores, hs-cTnT concentration, and integrated physician judgment

Two established risk scores were calculated: one exclusively using the 12-lead ECG (Better Analysis of ST-segment Elevations and Depressions in a 12-Lead-ECG score [BASEL score]), an automated score derived from quantitative ST-segment analysis of the digital ECG, and the recalibrated HEART score, embedded in the diagnostic pathway, a version adapted and validated to predict 30-day MACE, including AMI, combining clinical variables, the 12-lead ECG, and hs-cTn (available only in APACE).^25^, ^26^ For the direct comparison with hs-cTn, the currently best-validated and most widely used hs-cTn-assay was used: hs-cTnT-gen5. Hs-cTnT was measured at ED presentation using the Elecsys System with either the Modular Analytics E170 or the Cobas e602 (Roche Diagnostics) assay with a limit of detection of 5 ng/L, a 10% coefficient of variation at 13 ng/L, and the 99th percentile of a healthy reference population at 14 ng/L.^20^ Integrated physician judgment of the likelihood that an acute coronary syndrome (ACS) was the cause of the presenting symptoms was assessed by the treating ED physician 90 min after patient arrival using a visual analogue scale ranging from 0 (ACS certainly excluded) to 100 (ACS certain) with anchor points every 10%. At this time, the physician had reviewed all clinical information, the 12-lead ECG and the local hs-cTn concentration at ED presentation. This variable was collected prospectively only in the APACE study. The AI-ECG was applied retrospectively; physicians did not have access to it.

### Thresholds application: “10-min rule”

Thresholds from the original publication were applied to the computed predictions to evaluate the AI-ECG model’s direct rule-in and rule-out performance (referred to as the “10-min rule”). This initial classification was followed by the application of 0 h hs-cTnT thresholds recommended by the ESC (<5 ng/L: rule-out; 5–52 ng/L: observe zone; and ≥52 ng/L: rule-in) to mimic a real-world ED diagnostic pathway.^2^, ^3^ The pathway was applied in the population with available baseline hs-cTnT.

### Sensitivity analyses

Sensitivity analyses were conducted using alternative endpoints instead of the primary endpoint (AMI): STEMI; occlusion myocardial infarction (OMI; patients with an AMI and without coronary angiography were excluded); non-occlusive non-ST-segment elevation myocardial infarction (NSTEMI) (patients with STEMI or OMI, and those without coronary angiography, were excluded); and type 1 NSTEMI (patients with type 2 MI or STEMI were excluded). STEMI was defined according to the ESC criteria and the fourth UDMI as new ST-segment elevation at the J-point in ≥2 contiguous leads, or new left bundle branch block (LBBB) with ischaemic symptoms. OMI required an acute culprit lesion on invasive coronary angiography in at least one main coronary artery or their main branches with Thrombolysis in Myocardial Infarction (TIMI) flow 0–1, or TIMI flow 2 with >70% coronary stenosis and peak hs-cTnT >500 ng/L.^21^, ^27^

### Exploratory analyses

In a post hoc exploratory analysis, thresholds to rule in catheterisation laboratory activation, defined as a composite endpoint of STEMI or OMI, were derived and validated. Thresholds targeting a positive predictive value (PPV) of 70% for STEMI/OMI were derived in APACE-Light and validated in APACE. A second exploratory analysis was conducted to compare the performance of hs-cTnT thresholds derived from the ESC 0/1-h algorithm with those of the AI-ECG model.^15^ The overall agreement between the classification results of the two strategies was also computed. In a third exploratory analysis, if the original AI-ECG model triage thresholds did not achieve the clinically desired sensitivity of 99.0% for the rule-out of AMI, performance of lower triage thresholds was evaluated.^18^, ^19^, ^28^ In a fourth exploratory analysis, differences in the prevalence of type 2 MI between cohorts were adjusted for. Specifically, we performed bootstrap downsampling of type 2 MI cases in both cohorts to match the type 1 to type 2 MI ratio observed in the Korean development cohort (95:5). In each bootstrap iteration, Type 2 MI patients were randomly sampled to achieve the target ratio, and performance metrics were recalculated. In a fifth exploratory analysis, unspecified logistic regression models were derived incorporating the AI-ECG and the hs-cTnT in each of the datasets; the results are shown alongside the different comparators.

### Statistical analysis

Categorical variables were reported as count (percentage), and continuous variables as median (25th–75th percentiles [Q1-Q3]). Discrimination and precision were evaluated to assess the performance of the AI-ECG model and compare it with other biomarkers, scores and integrated physician diagnosis.^29^ Calibration intercept and calibration slope were reported. Model discrimination was quantified using the area under the receiver operating characteristic curve (AUC) and the area under the precision-recall curve (AUPRC), which summarises the trade-off between PPV and sensitivity.^30^ Confidence intervals for the AUC and their comparisons were estimated following DeLong’s method.^31^ Confidence intervals for the AUPRC, sensitivity, specificity, negative predictive value (NPV), and positive predictive value (PPV) were estimated using non-parametric bootstrap sampling (1000 samples), if not specified otherwise.^32^ The AUC of the AI-ECG model was further evaluated in prespecified subgroups: age (stratified by 75 years), sex, ECG device manufacturer (Schiller or Philips), time from chest pain onset to presentation, previous revascularisation, chronic kidney disease stage (CKD) defined as estimated glomerular filtration rate (eGFR) less than 60 mL/min/1.73 m^2^, left ventricular hypertrophy (LVH), atrial fibrillation (AF), LBBB, pacemaker rhythm, tachycardia. In APACE-Light the AF and LBBB features were retrieved using the open-access AI-ECG model from Ribeiro et al.^9^ Logistic regression models incorporating AI-ECG predictions and hs-cTnT were fitted using restricted cubic splines with three knots to allow for non-linear relationships, with knot placement left unspecified given the exploratory nature of the analysis.^6^ For the survival analysis, patients were grouped according to their AI-ECG score (low probability < 2.0%, intermediate probability 2.0–41.8%, and high probability ≥41.8%); the 5-year cumulative incidence of the composite of all-cause death or non-index AMI was plotted. The main statistical analysis plan was prespecified and agreed upon by the research institute and the company prior to the results. All statistical analyses were performed using R 4.5.1 (R Foundation for Statistical Computing, Vienna, Austria).

### Role of the funding source

The funders had no role in study design, data collection, data analysis, interpretation or writing of the report.

## Results

### Patient characteristics

A total of 17,397 patients were recruited (n = 9173 in APACE and n = 8224 in APACE-Light), of whom 12,630 were eligible for this analysis: 5047 in APACE and 7583 in APACE-Light (Supplemental Fig. S1A and B). Of these, 99.7% (12,596/12,630) also had hs-cTnT available at ED presentation (n = 5031 in APACE and n = 7565 in APACE-Light). The median age was 60 years (Q1–Q3: 47–73) in APACE and 58 years (Q1–Q3: 44–73) in APACE-Light, with 31% (1588/5047) and 39% (2941/7583) female participants, respectively (Table 1). Baseline characteristics were similar between all recruited patients and those included in the analysis except for a lower prevalence of dyslipidaemia (Supplemental Table S1). AMI was the centrally adjudicated diagnosis in 19% (968/5047) of patients in APACE (773 type 1 MI and 195 type 2 MI), including 6% (282/5047) with OMI, and in 18% (1386/7583) of patients in APACE-Light (18%; 1127 type 1 MI and 257 type 2 MI; 2 missing), including 6% (465/7583) with OMI.

**Table 1:** Baseline characteristics of the external validation cohorts.

	APACE	APACE Light
Demographics	**N = 5047**^a^	**N = 7583**^a^
Age (years)	60 (47, 73)	58 (44, 73)
Sex (Female)	1588 (31%)	2941 (39%)
Index presentation data		
ECG device manufacturer		
Philips	596 (12%)	847 (11%)
Schiller	4451 (88%)	6736 (89%)
History		
History of hypertension	2836 (56%)	3512 (46%)
Dyslipidaemia	2237 (44%)	2455 (32%)
Diabetes	752 (15%)	1233 (16%)
History of CAD	1538 (30%)	1943 (26%)
History of revascularisation	1319 (26%)	1603 (21%)
Vital parameters		
Systolic blood pressure (mm Hg)	139 (126, 155)	139 (125, 155)
Diastolic blood pressure (mm Hg)	82 (72, 91)	83 (75, 93)
Heart rate (beats per min)	72 (63, 83)	81 (71, 94)
Laboratory findings		
eGFR (mL/min per 1.73m^2^)	88 (71, 102)	94 (76, 108)
Initial hs-cTnT (ng/L)	8 (4, 20)	8 (4, 26)
AI-ECG		
AiTiA MI v2.00.00 probabilities	0.13 (0.04, 0.33)	0.11 (0.03, 0.31)
AiTiA MI v2.00.00 Decision		
Rule-out^b^	709 (14%)	1444 (19%)
Observe zone	3371 (67%)	4733 (62%)
Rule-in	967 (19%)	1406 (19%)
Outcomes		
Acute myocardial infarction	968 (19%)	1386 (18%)
Type 1 myocardial infarction	773 (15%)	1127 (15%)
Type 2 myocardial infarction	195 (4%)	257 (3%)
Non-ST-segment elevation myocardial infarction	814 (16%)	905 (12%)
ST-segment elevation myocardial infarction	154 (3%)	481 (6%)
Occlusion myocardial infarction	282 (6%)	465 (6%)
Unstable angina	377 (7%)	–
Non-cardiac or unknown cause of chest pain	26 (0.5%)	88 (1%)

a Median (Q1, Q3); n (%).

b The triage analyses were restricted to patients with hs-cTnT at ED presentation, in whom 706/5031 and 1437/7565 were ruled out and 966/5031 and 1401/7565 were ruled in.

### Diagnostic performance of the AI-ECG

The AI-ECG assigned predicted AMI probability ranging from 0 to 1. The model achieved a maximal score in a subset of AMI patients, demonstrating high diagnostic confidence in this subset (Fig. 1 A and D). The AUC for detecting AMI was 0.79 (95% CI: 0.77–0.80) in APACE and 0.84 (95% CI: 0.83–0.85) in APACE-Light (Supplemental Fig. S2B and E). Calibration showed overprediction among scores higher than 20%, confirmed by the intercept of both cohorts: −0.34 (95% CI: −0.43 to 0.25) in APACE and −0.41 (95% CI: −0.48 to 0.33) in APACE-Light (Supplemental Fig. S2C and F). The calibration slope was 0.66 (95% CI: 0.61–0.71) in APACE and 0.75 (95% CI: 0.71–0.79) in APACE-Light.

**Fig. 1:**
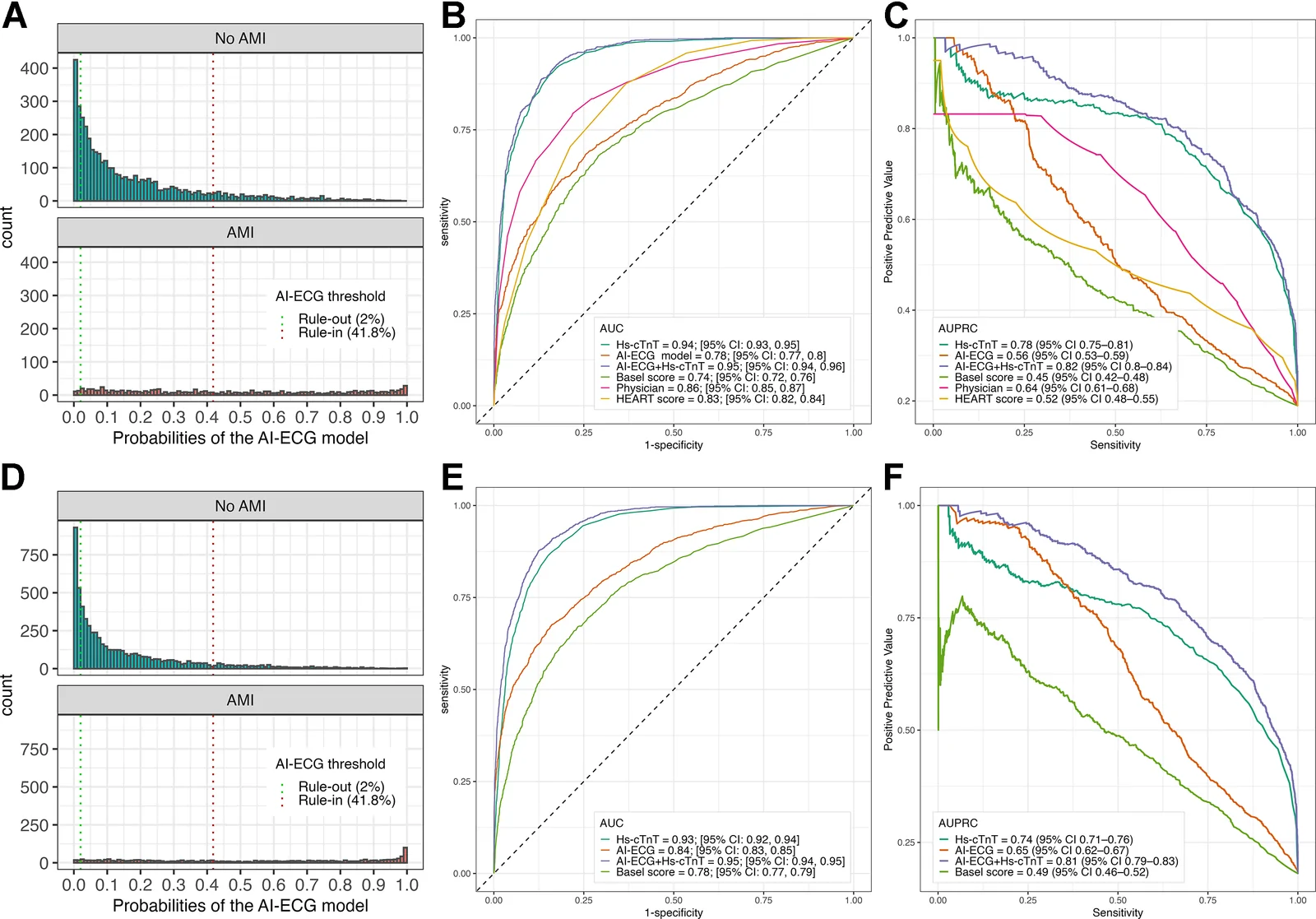
Predicted probability distribution of the AI-ECG model, receiver operating characteristic and precision–recall curves for acute myocardial infarction, comparing individual predictors and the incremental value of the AI-ECG model beyond high-sensitivity cardiac troponin T. Predicted probability distribution (A, D), Receiver operating characteristic curves (B, E) and precision–recall curves (C, F) showing the discrimination of high-sensitivity cardiac troponin T (hs-cTnT), the AI-ECG model, the Basel score, physician visual assessment scale, the HEART score, and the combination of the AI-ECG with hs-cTnT in the APACE cohort (B, C) and of hs-cTnT, the AI-ECG model, and the Basel score, and the combination of the AI-ECG with hs-cTnT in the APACE-Light cohort (E, F). AUC = Area under the Receiver-operating-characteristic curve; AUPRC = Area under the Precision-recall curve; AI-ECG = AiTiA MI v2.00.00; AMI: acute myocardial infarction; hs-cTnT = high-sensitivity cardiac troponin T.

In subgroup analyses, discrimination was lower in older patients (>75 years), early presenters (<3 and <6 h from chest pain onset), patients presenting with CKD, AF, LVH, tachycardia (only in APACE), with a history of revascularisation and, with a pacemaker rhythm (Supplemental Fig. S3). AI-ECG discrimination was also significantly lower in patients with LBBB (n = 185 [4%] and 327 [4%], AUC 0.73, [95% CI: 0.65–0.81] and AUC 0.68, [95% CI: 0.62–0.75]) than in patients without LBBB. Discrimination in women and men was similar (APACE: AUC 0.80 [95% CI: 0.77–0.83] versus 0.78 [95% CI: 0.76–0.80]; APACE-Light: AUC 0.84 [95% CI: 0.82–0.86] versus 0.84 [95% CI: 0.82–0.85]).

### Predefined thresholds application for AMI triage

When predefined thresholds were applied in APACE (n = 5031) (Fig. 2A, Tables 2 and 3), the AI-ECG model ruled out 14% (706/5031), achieving a sensitivity of 97.6% (95% CI: 96.5–98.5%) and an NPV of 96.7% (95% CI: 95.2–97.9%). In APACE-Light (n = 7565) (Fig. 2B, Tables 2 and 3), the model ruled out 19% (1437/7565) of patients, with a sensitivity of 97.5% (95% CI: 96.6–98.3%) and an NPV of 97.6% (95% CI: 96.8–98.4%). The characteristics of patients with missed AMI (false negatives) are summarised in Supplemental Table S2. In the APACE cohort, all 23 false negatives were NSTEMIs, and 57% (13/23) were identified as type 2 MI. Similarly, in APACE-Light, 91% (31/34) of the false negatives were NSTEMIs, 56% (19/34) were identified as type 2 MI. At the rule-in threshold (≥41.8%), the AI-ECG model ruled in 19% (966/5031) of patients in APACE and 19% (1401/7565) in APACE-Light, with a specificity of 88.3% (95% CI: 87.2–89.3%) and PPV of 50.8% (95% CI: 47.4–53.8%) in APACE, and a specificity of 90.3% (95% CI: 89.6–90.9%) and PPV of 57.1% (95% CI: 54.6–59.8%) in APACE-Light (Fig. 2A and B, Tables 2 and 3). The true positives for the combined STEMI and OMI endpoint, type 1 MI and type 2 MI were 245, 455 and 36 in APACE and 506, 727 and 73 in APACE-Light (Fig. 2A and B). The combined AI-ECG and hs-cTnT approach triaged 2877 patients (57%) to either rule-out (n = 1641/5,031, 33%) or rule-in (n = 1236/5,031, 25%), with a sensitivity of 97.5% (95% CI: 96.4–98.4%) and a specificity of 86.6% (95% CI: 85.6–87.6%) in APACE and triaged 4536 patients (60%) to either rule-out (n = 2597/7,565, 34%) or rule-in (n = 1939/7,565, 26%), with a sensitivity of 97.5% (95% CI: 96.5–98.2%) and a specificity of 86.5% (95% CI: 85.7–87.4%), in APACE-Light (Fig. 2A and B).

**Fig. 2:**
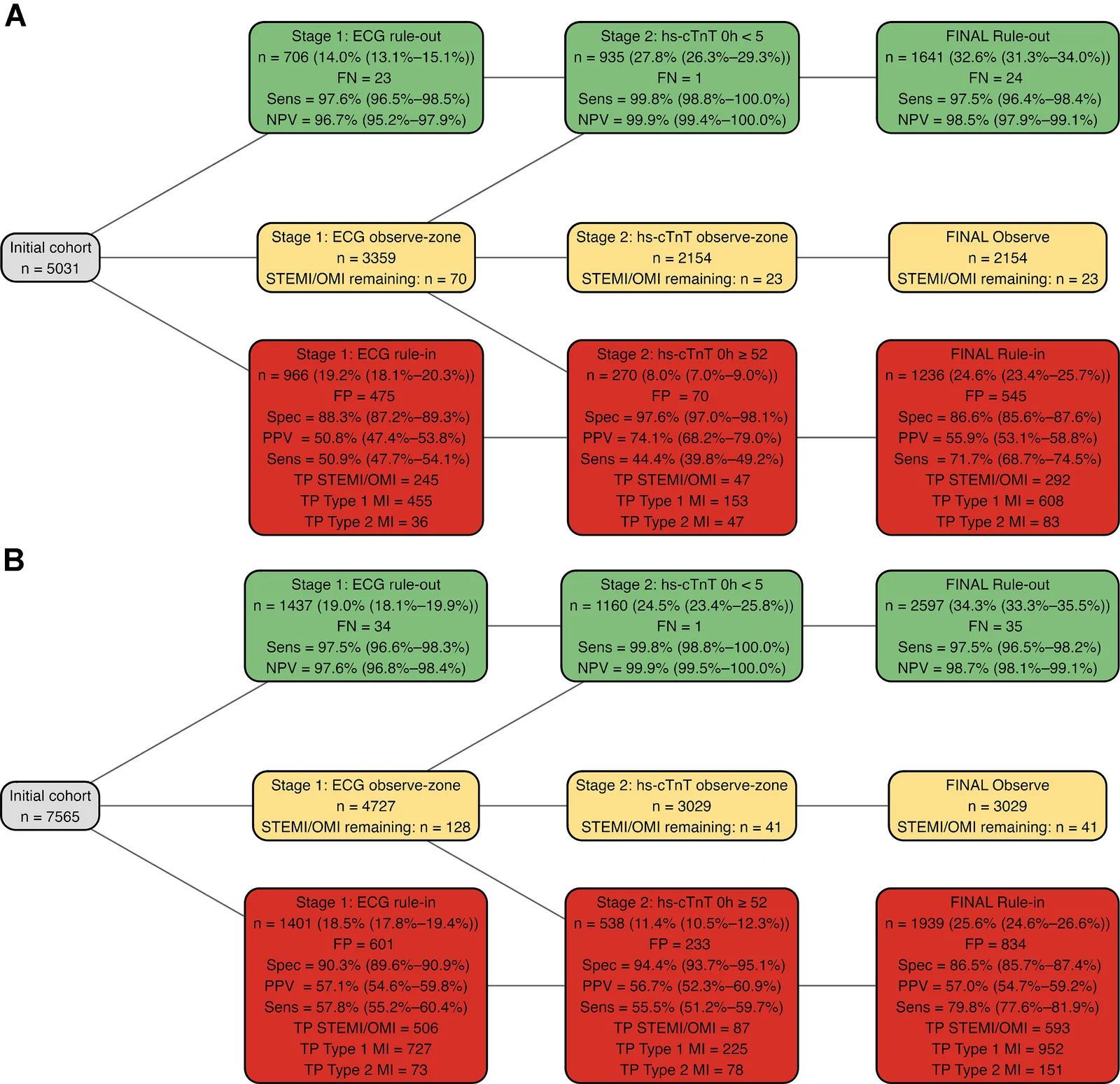
Diagnostic workflow applying the “10-min rule” and ESC 0/1-h algorithm baseline thresholds. (A) in APACE; (B) in APACE-Light. Patients were stratified according to their AI-ECG score (<2%: rule-out; 2–41.8%: observe zone; and ≥41.8%: rule-in) then by their initial high-sensitivity cardiac troponin T value (<5 ng/L: rule-out; 5–52 ng/L: observe zone; and ≥52 ng/L: rule-in) during their emergency department index visit. Sens = Sensitivity; Spec = Specificity; TP = True positives; STEMI = ST-segment elevation myocardial infarction; OMI = Occlusion myocardial infarction; MI = Myocardial infarction; FN = False negatives; NPV = Negative predictive value; FP = False positives; TP = True positives; PPV = Positive predictive value.

**Table 2:** AI-ECG model diagnostic safety and efficacy across AMI subtypes at the prespecified low-probability threshold (cutoff 2%).

Endpoints	APACE						APACE-Light					
	Sensitivity	Specificity	PPV	NPV	Rule-out Efficacy	FN	Sensitivity	Specificity	PPV	NPV	Rule-out Efficacy	FN
AMI	97.6	16.8	21.8	96.7	14.0	23	97.5	22.7	22.0	97.6	19.0	34
STEMI	100.0	14.5	3.6	100.0	14.0	0	99.4	20.2	7.8	99.8	19.0	3
OMI	99.6	15.5	6.9	99.9	14.6	1	99.4	20.7	7.8	99.8	19.4	3
Non-occlusive NSTEMI	96.6	16.7	15.5	96.9	14.8	22	96.2	22.1	12.6	98.0	20.2	28
Type 1 NSTEMI	98.4	16.8	15.2	98.6	14.8	10	98.0	22.7	11.9	99.1	20.7	13
Type 1 MI	98.7	16.3	17.5	98.6	14.0	10	98.7	22.1	18.1	99.0	19.0	15
Type 2 MI	93.3	14.3	4.2	98.2	14.0	13	92.6	19.4	3.9	98.7	19.0	19

All values (except FN) are in %. PPV = Positive predictive value; NPV = Negative predictive value; FN = False negatives.

**Table 3:** AI-ECG model diagnostic safety and efficacy across AMI subtypes at the prespecified high-probability threshold (cutoff 41.8%).

Endpoints	APACE						APACE-Light					
	Sensitivity	Specificity	PPV	NPV	Rule-in Efficacy	FP	Sensitivity	Specificity	PPV	NPV	Rule-in Efficacy	FP
AMI	50.9	88.3	50.8	88.4	19.2	475	57.8	90.3	57.1	90.5	18.5	601
STEMI	89.6	83.0	14.3	99.6	19.2	828	86.4	86.1	29.6	98.9	18.5	987
OMI	76.9	85.2	24.6	98.3	18.5	663	78.3	86.2	27.7	98.3	17.9	949
Non-occlusive NSTEMI	38.0	87.7	32.8	90.0	15.8	504	39.5	88.3	28.4	92.6	14.6	743
Type 1 NSTEMI	51.7	88.3	40.2	92.3	17.0	475	48.4	90.3	34.7	94.3	13.5	601
Type 1 MI	59.2	88.0	47.1	92.3	19.2	511	64.6	89.5	51.9	93.5	18.5	674
Type 2 MI	18.5	80.8	3.7	96.1	19.2	930	28.4	81.8	5.2	97.0	18.5	1328

All values (except FP) are in %. PPV = Positive predictive value; NPV = Negative predictive value; FP = False positives.

### Direct comparison with hs-cTnT, risk scores, and integrated clinical judgment

The diagnostic performance of the AI-ECG model for AMI was superior to that of the BASEL score in both cohorts. In APACE (restricted to the 5031 patients with hs-cTnT at presentation), the AI-ECG achieved an AUC of 0.78 (95% CI: 0.77–0.80) versus 0.74 (95% CI: 0.72–0.76, p < 0.001) for the BASEL score, and an AUPRC of 0.56 (95% CI: 0.53–0.59) versus 0.45 (95% CI: 0.42–0.48). Similarly, in APACE-Light, the AI-ECG outperformed the BASEL score with an AUC of 0.84 (95% CI: 0.83–0.85) versus 0.78 (95% CI: 0.77–0.79, p < 0.001), and an AUPRC of 0.65 (95% CI: 0.62–0.67) versus 0.49 (95% CI: 0.46–0.52) (Fig. 1 B, C, E, F).

The diagnostic performance of the AI-ECG model was inferior to hs-cTnT: AUC of 0.94 (95% CI: 0.93–0.95, p < 0.001) and AUPRC of 0.78 (95% CI: 0.75–0.81) in APACE, and AUC of 0.93 (95% CI: 0.92–0.94, p < 0.001) and AUPRC of 0.74 (95% CI: 0.71–0.76) in APACE-Light. The recalibrated HEART score had an AUC of 0.83 (95% CI: 0.82–0.84) and an AUPRC of 0.52 (95% CI: 0.48–0.55) in APACE. Integrated physician judgment available at 90 min after ED presentation had higher diagnostic performance than the AI-ECG, with an AUC of 0.86 (95% CI: 0.85–0.87, p < 0.001) and an AUPRC of 0.64 (95% CI: 0.61–0.68) in APACE.

The AI-ECG model showed some statistically significant incremental value when combined with hs-cTnT in logistic regression compared with hs-cTnT alone. The combined model achieved an AUC of 0.95 (95% CI: 0.94–0.96) and an AUPRC of 0.82 (95% CI: 0.80–0.84) in APACE and an AUC of 0.95 (95% CI: 0.94–0.95) and an AUPRC of 0.81 (95% CI: 0.79–0.83) in APACE-Light (all p < 0.001, Fig. 1 B, C, E, F). Decision curve analysis confirmed the superiority of the combined model (Supplemental Fig. S4).

### Sensitivity analyses for other endpoints

Among the single predictors, the AI-ECG model achieved the highest diagnostic performance for STEMI, with AUCs of 0.94 (95% CI: 0.92–0.96) and 0.94 (95% CI: 0.93–0.95) and AUPRCs of 0.58 (95% CI: 0.50–0.66) and 0.69 (95% CI: 0.65–0.73) in APACE and APACE-Light, respectively (Fig. 3 B, C, E, F). The AI-ECG model showed AUCs of 0.90 (95% CI: 0.88–0.92) and 0.91 (95% CI: 0.89–0.92) and AUPRCs of 0.55 (95% CI: 0.48–0.60) and 0.54 (95% CI: 0.49–0.59) for the diagnosis of OMI, in APACE and APACE-Light, respectively (Fig. 4 B, C, E, F). AI-ECG probability and ECG-based rule-in rates were consistently higher for TIMI 0/1 than TIMI 2 flow in both cohorts (APACE: 77.3% (n = 207) versus 66.4% (n = 140); APACE-Light: 80.4% (n = 363) versus 63.9% (n = 155)) (Supplemental Tables S3 and S4). The AI-ECG model showed AUCs of 0.76 [95% CI: 0.74–0.79] and 0.78 [95% CI: 0.76–0.81] for the diagnosis of non-occlusive NSTEMIs, with AUPRCs of 0.28 [95% CI: 0.24–0.33] and 0.29 [95% CI: 0.24–0.32] (Supplemental Fig. S5). The AI-ECG model showed AUCs of 0.80 [95% CI: 0.78–0.82] and 0.82 [95% CI: 0.80–0.83] for the diagnosis of type 1 NSTEMIs, with AUPRCs of 0.45 [95% CI: 0.41–0.49] and 0.38 [95% CI: 0.34–0.42] (Supplemental Fig. S6). The safety and efficacy for each of the endpoints are summarised in the Table 2 for the low-risk threshold and Table 3 for the high-risk threshold.

**Fig. 3:**
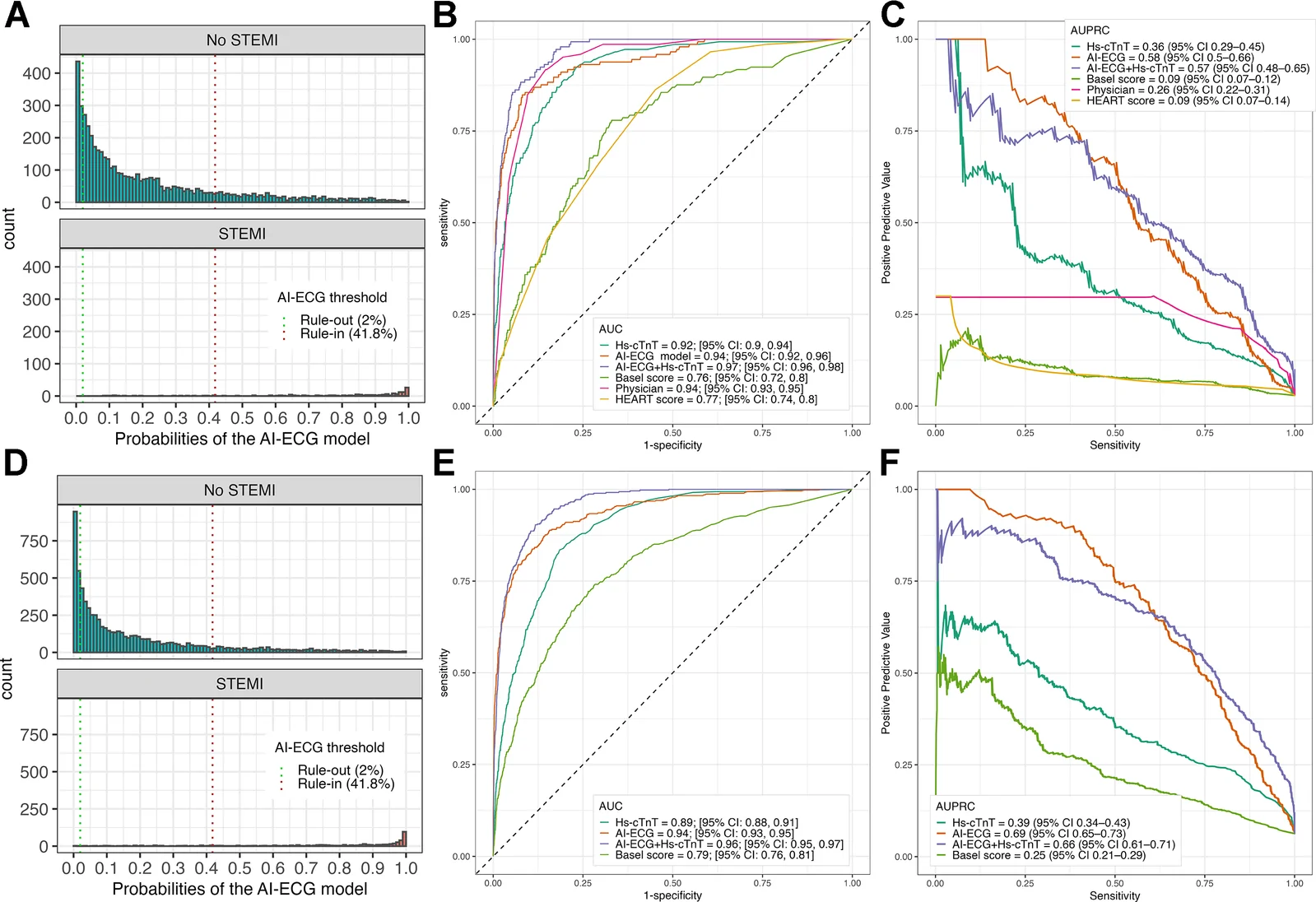
Predicted probability distribution, receiver operating characteristic and precision–recall curves of the AI-ECG model for ST segment elevation myocardial infarction, comparing individual predictors and the incremental value of the AI-ECG model beyond high-sensitivity cardiac troponin T. Predicted probability distribution (A, D), Receiver operating characteristic curves (B, E) and precision–recall curves (C, F) showing the discrimination of high-sensitivity cardiac troponin T (hs-cTnT), the AI-ECG model, the Basel score, physician visual assessment, the HEART score, and the combination of the AI-ECG with hs-cTnT in the APACE cohort (B, C) and of hs-cTnT, the AI-ECG model, the Basel score, and the combination of the AI-ECG with hs-cTnT in the APACE-Light cohort (E, F). AUC = Area under the Receiver-operating-characteristic curve; AUPRC = Area under the Precision-recall curve; AI-ECG = AiTiA MI v2.00.00; AMI: acute myocardial infarction; hs-cTnT = high-sensitivity cardiac troponin T.

**Fig. 4:**
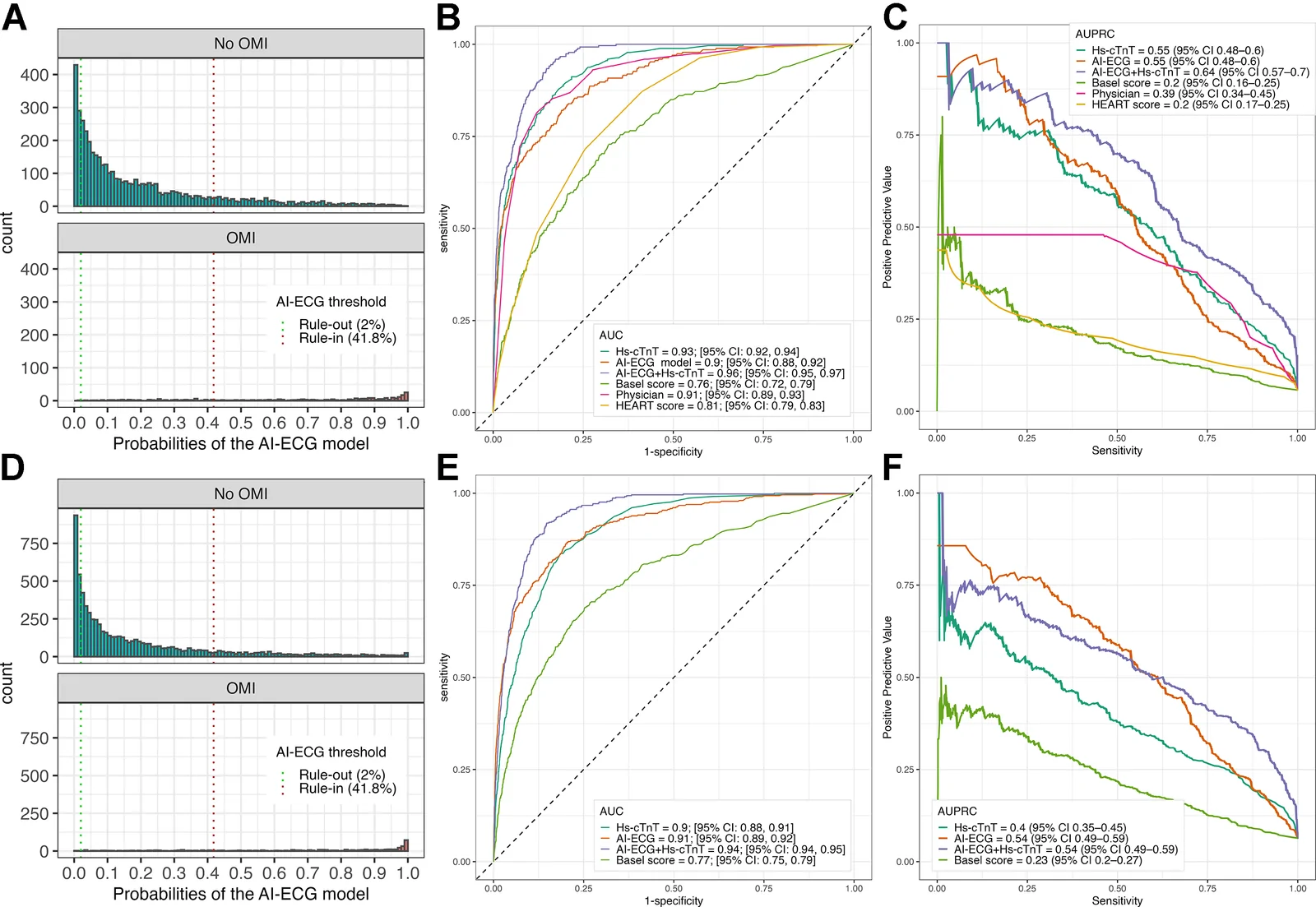
Predicted probability distribution receiver operating characteristic and precision–recall curves of the AI-ECG model, for occlusion myocardial infarction, comparing individual predictors and the incremental value of the AI-ECG model beyond high-sensitivity cardiac troponin T. Predicted probability distribution (A, D), Receiver operating characteristic curves (B, E) and precision–recall curves (C, F) showing the discrimination of high-sensitivity cardiac troponin T (hs-cTnT), the AI-ECG model, the Basel score, physician visual assessment, the HEART score, and the combination of the AI-ECG with hs-cTnT in the APACE cohort (B, C) and of hs-cTnT, the AI-ECG model, the Basel score, and the combination of the AI-ECG with hs-cTnT in the APACE-Light cohort (E, F). AUC = Area under the Receiver-operating-characteristic curve; AUPRC = Area under the Precision-recall curve; AI-ECG = AiTiA MI v2.00.00; AMI: acute myocardial infarction; hs-cTnT = high-sensitivity cardiac troponin T.

### Long term outcomes

Over 5 years of follow-up, the cumulative incidence of AMI or all-cause death was highest in patients classified as rule-in, intermediate in those assigned to observe, and lowest in the rule-out group. Using the ECG model, over the 5-year follow-up, the cumulative incidence was 25.9% in the rule-in group and 17.2% in the observe group compared with 4.5% in the rule-out group. Similarly, with the hs-cTnT-based thresholds, the cumulative incidence was 36.9%, 19.7%, and 2.2%, respectively (Supplemental Fig. S7).

### Exploratory analyses

An AI-ECG probability ≥79% identified 6.5% (495/7565) of patients as rule-in positive in the derivation cohort (APACE-Light) and 5.5% (277/5031) in the validation cohort (APACE), with a specificity for STEMI/OMI of 97.9% and 97.6% and a PPV for STEMI/OMI of 70.3% (95% CI: 66.1–74.3) and 58.5% (95% CI: 52.4–64.3), respectively (Fig. 5). In APACE, after the first hs-cTnT threshold, PPV for AMI reached 79.5% (95% CI: 76.4%–82.3%) while sensitivity reached 60.6% (57.4–63.7%). The overall agreement between the AI-ECG model and the hs-cTnT thresholds recommended by the ESC 0/1-h algorithm was 58% (n = 2898) and 60% (n = 4547) in the APACE and APACE-Light cohorts, respectively (Supplemental Fig. S8). As the original AI-ECG model triage thresholds did not achieve the clinically desired high sensitivity of 99.0% for the rule-out of AMI, we evaluated the performance of lower triage thresholds (Supplemental Fig. S9). No threshold achieved sensitivity and NPV exceeding 99.0% for AMI detection. For STEMI/OMI, sensitivity and NPV remained above 99.5% and 99.0%, respectively, in both datasets. When the Type 2 MI population was downsampled to match the development cohort ratio of 5% among patients with AMI, the overall safety of the AI-ECG model increased, with a reduction in false-negative patients (Supplemental Fig. S10).

**Fig. 5:**
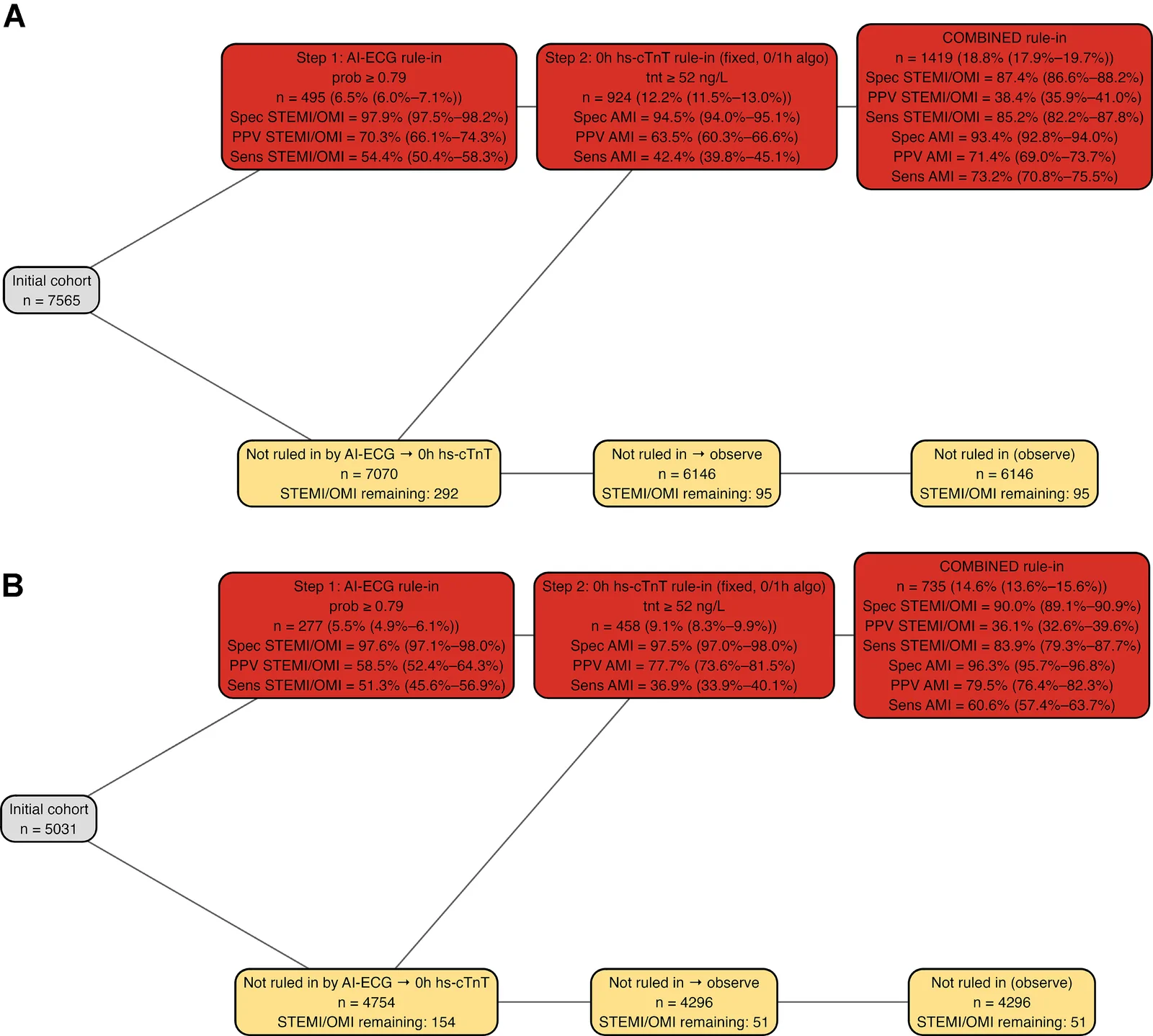
Derivation (A) and validation (B) of a diagnostic workflow using the AI-ECG to rule-in ST-segment elevation myocardial infarction or occlusion myocardial infarction before applying the ESC0/1-h algorithm for detecting acute myocardial infarction. (A) APACE-Light (B) APACE. STEMI = ST-segment elevation myocardial infarction; OMI = Occlusion myocardial infarction; AMI = Acute myocardial infarction; FN = False negatives; NPV = Negative predictive value; FP = False positives; PPV = Positive predictive value; Sens = Sensitivity; Spec = Specificity.

## Discussion

In this external validation study using two large prospective European cohorts, we evaluated the diagnostic performance and generalizability of the recently developed AI-ECG model based on the first 12-lead ECG available at ED presentation in patients presenting with symptoms suggestive of AMI. The AI-ECG was directly compared with the contemporary standards of care, including risk scores, hs-cTnT, and integrated physician judgment. We report eight major findings.

**First**, the AI-ECG model showed good discrimination for AMI in both cohorts (AUC 0.79 and 0.84), with overall acceptable calibration. **Second**, the AI-ECG model was statistically superior to the BASEL score, but inferior to hs-cTnT at ED presentation, the recalibrated HEART score, and integrated physician judgment. **Third,** the AI-ECG model provided some incremental diagnostic value when combined with hs-cTnT statistically significantly further increasing the AUC at ED presentation. **Fourth,** the AI-ECG model, as a standalone tool, applying predefined thresholds for the rule-out of AMI achieved a sensitivity of 97.6% and 97.5% and rule out 706 (14.0%) and 1437 (19.0%) of patients in APACE and APACE-light, respectively. Most missed patients had adjudicated type 2 NSTEMI. **Fifth**, a combined strategy using AI-ECG and established 0 h hs-cTnT cutoffs recommended for the early rule-out (<5 ng/L) and early rule-in (≥52 ng/L) triaged 2877 patients (57.2%) to either rule-out (n = 1641, 32.6%) or rule-in (n = 1236, 24.6%) with a sensitivity of 97.5% and a specificity of 86.6% in APACE. Similarly, the combined approach triaged 4536 patients (60.0%) to either rule-out (n = 2597, 34.3%) or rule-in (n = 1939, 25.6%) with a sensitivity of 97.5% and a specificity of 86.5% in APACE-Light. **Sixth**, discrimination was excellent for STEMI (AUC 0.94) and OMI (AUC 0.90 and 0.91). **Seventh**, a newly derived rule-in threshold identified STEMI/OMI patients with high precision while maintaining high sensitivity for AMI within the combined hs-cTnT pathway. **Eighth**, the AI-ECG model provided long-term prognostic information beyond the index ED visit for the composite of all-cause death or non-index AMI over 5 years.

These findings extend prior pioneering studies on ECG-based DL models for patients presenting to the ED with suspected AMI.^15^, ^33^, ^34^, ^35^, ^36^ Most previous models were developed using large retrospective national cohorts, often derived from electronic health records, and commonly trained on all available ECGs from consecutive patients, including pre-hospital recordings. This approach reduces sampling bias by increasing dataset heterogeneity.^33^, ^34^ However, it provides limited guidance for clinical implementation. To our knowledge, this is the first external validation of this AI-ECG model in two large European prospective cohorts, encompassing multiple ECG device manufacturers.

This study has important clinical implications, since the AI-ECG model maintained good, but not very good performance across cohorts with a lower AUC than that reported in the Korean population (0.79 and 0.84 versus 0.91).^15^ It was also the first including a direct comparison with hs-cTnT, used as recommended in current guidelines, and as a continuous (and not binary) variable.^2^, ^3^, ^6^ Direct comparison of AI-ECG and hs-cTnT showed inferiority of AI-ECG. Consistent with this, the predefined AI-ECG thresholds for ruling-out AMI achieved a sensitivity of 97.5–97.6%, below the 99% sensitivity target used to derive these thresholds. A definite rule-out decision could be made in only one in seven (APACE) to one in five (APACE-Light) patients, with the remaining patients requiring further evaluation. However, the acceptable trade-off between sensitivity (safety) and triage efficiency ultimately depends on local risk tolerance. Individual institutions may consider a sensitivity of 97.5% for AMI overall, combined with a sensitivity above 99% for STEMI/OMI, sufficiently safe for ruling out patients. Notably, sensitivity increased when we downsampled the cohort to match the Type 1:Type 2 MI ratio reported in the Korean development cohort, indicating that our estimate is case-mix dependent and may achieve safer results in a setting with lower proportion of Type 2 MI.

The AI-ECG showed excellent discrimination for STEMI (AUC 0.94) and OMI (AUC 0.90 and 0.91 in APACE and APACE-Light, respectively). In a post hoc exploratory analysis, a threshold on the model's original AMI probability was derived in APACE-Light and validated in APACE to rule in a composite STEMI/OMI endpoint, targeting 70% PPV. In the validation, this strategy ruled in 40% fewer patients than the main analysis (735 versus 1236) demonstrating an increase in PPV for AMI from 55.9% to 79.5%, at the cost of an 11-point drop in sensitivity (60.6% versus 71.7%), a trade-off appropriate for a rule-in strategy, which favours precision over sensitivity. This suggests AI-ECG could help triage patients toward very early (e.g., within 2 h) coronary angiography, a hypothesis warranting prospective testing.^37^

Despite possible application for the early rule-in of STEMI/OMI, our exploratory analysis suggests that the AI-ECG model may be better suited as an adjunct to hs-cTnT at ED presentation rather than as a standalone decision-making tool for AMI. This is in line with existing machine-learning tools for suspected MI, such as MI3, CoDE-ACS, ARTEMIS, and more recently FAST-NSTEMI, which similarly combine hs-cTn with clinical variables, including the ECG, rather than relying on either alone.^16^, ^17^, ^38^, ^39^ Further research on optimally combining AI-ECG with hs-cTn and clinical variables is warranted. Relatedly, the platform provided by the developing team outputs a probability without possibility of identifying the underlying ECG features related to it. This lack of explainability may limit standalone adoption and warrants dedicated interpretability work by the model's developers, as clinicians may favour explainable models of comparable performance.

The lower performance observed in older patients, and those with a higher comorbidity burden, raises concerns about populations typical of pre-hospital and primary-care settings. Our findings therefore support possible diagnostic validity within the ED but do not justify out-of-hospital use. Ambulance and primary-care application would likely require recalibration and prospective evaluation before implementation. Women accounted for fewer patients (4529 [36%] of 12,630), as is typical of chest pain cohorts. Although AI-ECG discrimination was similar between sexes, women accounted for 32 (56%) of the 57 false negatives. This finding should be interpreted cautiously given the small number of false negatives.

Differences in the proportion of type 1 MI to type 2 MI between the recent Korean development cohort (95:5) and these two external validation cohorts (80/20 and 81/19) emerged as an important contributor to the differences in diagnostic performance of the AI-ECG, as shown in subgroup and exploratory analyses that downsampled the proportion of type 2 MI. The proportion of STEMI within AMI also differed (40.4% in the Korean cohort versus 15.9% and 34.7% in APACE and APACE-Light, respectively), partly attributable to enrolment differences such as the requirement for informed consent. Beyond cohort composition, differences in comorbidity burden, differing interpretation of the Universal Definition of MI during central adjudication (by ED physicians in the Korean study versus in ours), and sources of ECG-signal noise may also have contributed to the lower performance.

Several limitations merit consideration when interpreting these findings. First, although AMI was centrally adjudicated according to the UDMI using extensive patient documentation including cardiac imaging, the 12-lead ECG, serial hs-cTn measurements, and clinical follow-up, a small number of misclassifications may have occurred. While these may have led to an underestimation of the point estimate for the AUC of AI-ECG, they would not have affected the relative performance of the direct comparators among each other and therefore the inferiority of AI-ECG versus hs-cTnT, the recalibrated HEART score, and integrated physician judgment. Second, it is important to acknowledge that central adjudication included pre-hospital ECG recordings, if performed. This enabled e.g., the detection of STEMI even if significant ST-segment elevation was present only in the pre-hospital ECG, but no longer in the ECG at ED presentation. However, the possible availability of a preclinical ECG disadvantages the AI-ECG model versus the central adjudicators only for the diagnosis of STEMI and also does not disadvantage AI-ECG in its direct comparison versus hs-cTnT. Moreover, the present analysis relied on a single ECG at ED presentation while serial in-hospital ECGs could capture dynamic changes and further improve performance, which warrants evaluation in future studies. Third, APACE and APACE-Light were conducted in patients presenting to the ED. Therefore, we cannot comment on the performance of the AI-ECG in other clinical settings. Fourth, the UDMI defines NSTEMI diagnosis based on cardiac troponin dynamics combined with at least one supporting criterion (symptoms, ECG changes, imaging, or angiographic evidence), of which ECG change is not a requirement. This may favour cardiac troponin-based predictors over ECG-based predictors. Fifth, as this diagnostic study used centrally adjudicated AMI as the reference standard, we could not evaluate implementation barriers or the downstream medical and economic impact of the AI-ECG model’s use in real clinical practice. Sixth, both validation cohorts were European, and the generalizability of our findings to other populations remains to be determined. Seventh, despite the promising performance of the derived rule-in thresholds, the limited interpretability of the AI-ECG may hinder clinical adoption of the validated tool. Eighth, the prevalence of AMI was high in both cohorts, which may have led to an overestimation of PPV relative to lower-prevalence settings. However, AMI prevalence (19% and 18%) was comparable to that of the Korean development cohort (22.8%), thereby limiting the risk of bias due to prevalence shift.

In conclusion, the AI-ECG provided high, but not very high, diagnostic accuracy for AMI. Its main value may lie in ruling in patients: a STEMI/OMI-specific threshold precisely identified a small group who could be triaged toward very early coronary angiography. However, as a diagnosis of AMI is unlikely to be ruled out on ECG alone in current practice, AI-ECG may be regarded as an adjunct to hs-cTnT rather than a standalone tool. These findings warrant development of a combined model and prospective implementation studies to confirm clinical benefit — e.g., improved patient outcomes, safer triage, and more appropriate catheterisation laboratory activation.

## Contributors

AC, MSL, PL-A, IS, and CM conceived the study and its design. IS, PC, and CM acted as supervisors for the study. AC performed the external validation in Basel under the supervision of IS and CM, who were responsible for data collection in the APACE and APACE-Light cohorts. MSL, Y-YJ, KK, KSK, SY, HSL, and J-mK developed the artificial intelligence–based ECG algorithm and provided insights and guidance on its implementation and interpretation. JB, LK, PB, LC, TZ, EK, DMG, LH-R, GH, T-YT, MN, MC, NG, OM, KS, RB, and CN were involved in data collection and curation, and contributed to data interpretation. CM, OM, MC, RB, CN, KS, PC, FM, and J-mK provided resources for the study. AC, PL-A, JB, TZ, LK, EK, PB, LH-R, GH, IS, and CM had access to and verified the raw data. AC and PL-A performed the data analysis. IS validated the analysis. AC, MSL, PL-A, IS, and CM interpreted the data. AC, PL-A, IS, and CM drafted the manuscript. All authors had full access to the data presented in the study. All authors revised and edited the manuscript critically for important intellectual content. All authors provided their final approval of the version to be published. All authors had final responsibility for the decision to submit for publication. APACE and APACE-Light investigators contributed to data generation.

## Data sharing statement

Requests for the statistical analysis plan for academic non-profit purposes can be sent to arnaudtanguy.champetier@usb.ch. The AiTiA MI v2.00.00 model/API used in this study is proprietary to Medical AI Co., Ltd. (Seoul, Republic of Korea). Access to the model was granted under a research agreement that does not permit sharing of the model, or data generated with it, with third parties. Requests for access to the model within a research collaboration can be sent to lylm@medicalai.com.

## Declaration of interests

MSL, KSK, HSL, and SY are employees of Medical AI Co., Ltd., the manufacturer of the AI-ECG algorithm evaluated in this study, provided the access to the AI-ECG algorithm/model, and hold stock or stock options in the company. Y-YJ is an employee and Executive Director of Medical AI Co., Ltd. and holds stock options in the company. J-mK is the chief executive officer and founder of Medical AI Co., Ltd. and holds stock in the company. KK holds stock of Medical AI Co., Ltd. PL-A reports a research grant from the Swiss Heart Foundation (FF24149), consulting fees from bioMérieux as a scientific and statistical expert, and speaker honoraria from Quidel, Roche Diagnostics, and Polymedco, all outside the submitted work. CM reports grants or contracts from AstraZeneca, bioMérieux, Boehringer Ingelheim, MSD, Novartis, Novo Nordisk, and Sphingotec; consulting fees from Abbott, Boehringer Ingelheim, Daiichi Sankyo, Idorsia, MSD, Novo Nordisk, Roche, and SpinChip; and payment or honoraria for lectures from Abbott, AstraZeneca, Bayer, Boehringer Ingelheim, BMS, Eli Lilly, Novartis, Novo Nordisk, Roche, Sanofi, and Siemens; all paid to the institution and all outside the submitted work. JB reports research grants from the University of Basel, the University Hospital Basel, the Division of Internal Medicine, the Swiss Academy of Medical Sciences, the Gottfried and Julia Bangerter-Rhyner Foundation, the Swiss National Science Foundation, and the Freie Akademische Gesellschaft Basel; consulting fees and honoraria for lectures from Siemens, Roche Diagnostics, Ortho Clinical Diagnostics, Quidel Corporation, Beckman Coulter, and Terumo; and support for attending meetings and travel from Medtronic, Cordis, and VascularMedical, all paid to the institution and outside the submitted work; JB also declares participating on a data safety monitoring board for the TARGET-CTCA trial without payment. LK reports research grants from the Swiss Heart Foundation, the University of Basel, the Swiss Academy of Medical Sciences, the Gottfried and Julia Bangerter-Rhyner Foundation, and the Freiwillige Akademische Gesellschaft Basel, and speaker honoraria from Dr Risch, Polymedco, Roche Diagnostics, Abbott, and Siemens, all outside the submitted work. EK reports grants from the Swiss Heart Foundation, the Mach Gaensslen Stiftung, the Propatient Stiftung Basel, the Freiwillige Akademische Gesellschaft Basel, and the Bangerter-Rhyner Foundation; consulting fees from SpinChip; payment or honoraria for lectures from Medtrix AG Basel, the Solothurner Spitäler, and the Seniorenuniversität Basel, Bern, and Zurich; and payment for expert testimony from Vifor Pharma, all outside the submitted work. PB reports consulting fees from Aurevia, paid to him, outside the submitted work. NG reports a grant from the Stiftung für Klinische Forschung, Luzerner Kantonsspital (P-2025-06), providing protected research time, outside the submitted work. OM reports payment for expert testimony from Gilead, regarding HIV detection in emergency departments, and from bioMérieux, regarding point-of-care troponin, outside the submitted work. FM reports grants from the Deutsche Forschungsgemeinschaft (SFB TRR219, Project-ID 322900939) and the Deutsche Herzstiftung; consulting fees, payment or honoraria for lectures, and support for attending meetings and travel from Ablative Solutions, AstraZeneca, Inari, Medtronic, Merck, Novartis, Philips, and ReCor Medical, all received until May 2024; scientific support received by his previous institution, Saarland University, from Ablative Solutions, Medtronic, and ReCor Medical; and an unremunerated board membership of the European Society of Cardiology, all outside the submitted work. All other authors declare no competing interests relevant to this publication.
