# Supplementary material for: External validation of an artificial intelligence–based ECG algorithm for the early diagnosis of acute myocardial infarction: a prospective diagnostic cohort study
Source: Lancet Reg Health Eur. 2026 Nov 7;71:101912. doi: 10.1016/j.lanepe.2026.101912 (PMC13769588; doi:10.1016/j.lanepe.2026.101912)
Supplement: Supplementary file 1 [file mmc1.docx]

SUPPLEMENTAL MATERIAL

**External Validation of an Artificial Intelligence–based ECG Algorithm for the Early Diagnosis of Acute Myocardial Infarction: A prospective diagnostic cohort study**

**Supplemental Methods**

**Model Overview and Development Data**

**Model Architecture and Mechanism**

**Input and Output Specifications**

**Validation and Clinical Performance**

**Regulatory Approval Status**

**Supplemental Tables**

**Supplemental Table 1 – Baseline characteristics of all recruited patients and those included in the analysis**

**Supplemental Table 2 – Patients With an Adjudicated Diagnosis of AMI Missed (False Negative) by the AI-ECG model**

**Supplemental Table 3 – AMI-diagnosed patients: TIMI 0/1 vs TIMI 2 (TIMI 3 for reference)**

**Supplemental Table 4 – TIMI flow distribution among patients with STEMI**

**Supplemental Figure Legends**

**Supplemental Figure 1 – Patient flowcharts of the (A) APACE and (B) APACE-Light cohorts**

**Supplemental Figure 2 – Diagnostic performance of the AI-ECG model for the detection of AMI in the APACE and APACE-Light cohorts**

Distribution of probabilities across patients with and without acute myocardial infarction (AMI) in the APACE (A) and the APACE-Light (D) cohorts; Receiver-operating-characteristic curve showing the discrimination of the AI-ECG model in the APACE (B) and APACE-Light (E) cohorts; Calibration curve of the AI-ECG model predictions, assessing the agreement between predicted outcomes and observed proportion in the APACE (C) and APACE-Light (F) cohorts, the red line represents optimal calibration, the green line represents the sigmoid calibration, the black solid line represents the Loess based calibration curve, and the black dashed line represents the 95% confidence interval of the calibration curve.

AUC= Area under the Receiver-operating-characteristic curve; AI-ECG=AiTiA MI v2.00.00; AMI: acute myocardial infarction.

**Supplemental Figure 3 – Diagnostic performance of the AI-ECG model across patient subgroups in (A) APACE and (B) APACE-Light**

AUC=area under the receiver operating characteristic curve; CI=confidence intervals; AI-ECG=AiTiA MI v2.00.00; eGFR= estimated glomerular filtration rate; LVH=left ventricular hypertrophy; AF=atrial fibrillation; LBBB=left bundle branch block.

**Supplemental Figure 4 – Decision curve analysis performance of AiTiAMI score thresholds for rule-out and rule-in of acute myocardial infarction in APACE (A) and APACE-Light (B)**

hs-cTnT=high-sensitivity cardiac troponin T; AI-ECG=AiTiA MI v2.00.00

**Supplemental Figure 5 – Diagnostic and precision performance of the AI-ECG model for non-occlusive NSTEMI diagnosis comparing individual predictors and the incremental value of the AI-ECG model beyond high-sensitivity cardiac troponin T, in (A–C) APACE and (D–F) APACE-Light**

AUC=area under the receiver operating characteristic curve; AUPRC=area under the precision–recall curve; AI-ECG=AiTiA MI v2.00.00; hs-cTnT=high-sensitivity cardiac troponin T; NSTEMI=non-ST-segment elevation myocardial infarction.

**Supplemental Figure 6 – Diagnostic and precision performance of the AI-ECG model for type 1 NSTEMI diagnosis in (A–C) APACE and (D–F) APACE-Light**

AUC=area under the receiver operating characteristic curve; AUPRC=area under the precision–recall curve; AI-ECG=AiTiA MI v2.00.00; hs-cTnT=high-sensitivity cardiac troponin T; NSTEMI=non-ST-segment elevation myocardial infarction.

**Supplemental Figure 7 – 5-years all-cause death or non-index acute myocardial infarction using (A) the AI-ECG model and (B) high-sensitivity cardiac troponin T**

Kaplan–Meier and cumulative incidence function plot depicting 5-years cumulative incidence in patients with chest pain stratified according to their AI-ECG score (A) (<2: rule-out; 2–41.8: observe-zone; and ≥41.8: rule-in) and by their initial high-sensitivity cardiac troponin T value (B) (<5 ng/L: rule-out; 5-52 ng/L: observe-zone; and ≥52 ng/L: rule-in) during their emergency department index visit.

**Supplemental Figure 8 – Classification agreement between AI-ECG model decision thresholds and baseline high-sensitivity cardiac troponin T thresholds of the European Society of Cardiology 0/1-hour algorithm in (A) APACE and (B) APACE-Light**

Green zones correspond to rule-out patients, orange zones to the observe zone, and red zones to rule-in patients.

FN=false negatives; NPV=negative predicted value; Sens=sensitivity; FP=false positives; PPV=positive predicted value; Spec=Specificity.

**Supplemental Figure 9 – Clinical utility and diagnostic performance of AiTiAMI score thresholds for rule-out and rule-in of acute myocardial infarction and a combined ST elevation myocardial infarction and occlusion myocardial infarction endpoint in APACE (A) and APACE-Light (B)**

NPV=negative predicted value; PPV=positive predicted value; STEMI=ST elevation myocardial; OMI=occlusion myocardial infarction

**Supplemental Figure 10 – Bootstrapped diagnostic workflow using the 10-minute rule and ESC 0/1-h thresholds with Type 2 MI representing 5% of the acute myocardial infarctions**

Green zones correspond to rule-out patients, orange zones to the observe zone, and red zones to rule-in patients.

FN=false negatives; NPV=negative predicted value; Sens=sensitivity; FP=false positives; PPV=positive predicted value; Spec=Specificity.

**Model Overview and Development Data**

AiTiA MI version 2.00.00 (Medical AI Co., Ltd., Seoul, Republic of Korea) is an advanced AI/ML-enabled Software as a Medical Device (SaMD) for the early identification of acute myocardial infarction (AMI).

- Target Population: Adult patients suspected of having acute MI.
- Development Dataset: A total of 40,165 ECGs from 28,596 patients across 16 large-scale hospitals. [7,8]
- Disease Definition: Acute MI defined by the 4th Universal Definition of MI (2018).

**Model Architecture and Mechanism**

The model utilizes a hybrid architecture engineered to capture both high-dimensional local morphology and global temporal context.[1]

- Foundation Model: Pre-trained using masked signal reconstruction on a large-scale self-supervised dataset of 1.29 million ECGs, enabling robust cardiac representation learning. [1-6]
- Hybrid Architecture - Convolutional Encoder (ResNet): Extracts high-dimensional features of local waveforms (e.g., P-wave, QRS complex, T-wave)
- Hybrid Architecture - Transformer-based Encoder: Utilizes multi-head attention to model long-range temporal dependencies and global rhythm characteristics across the entire recording.
- Multi-task Learning: Simultaneously predicts the probability of AMI (primary task) and detects ST-elevation Myocardial Infarction (STEMI) (auxiliary task), enhancing sensitivity to acute ischemic changes.

**Input and Output Specifications**

- Input: Raw 12-lead ECG data (10s duration, 500Hz sampling rate) in XML format.
- Output - Probability Score: Continuous value (0–100).
- Output - Risk Levels: High (Score >= 41.8), Intermediate (2.0–41.7), Low (Score < 2.0).

**Validation and Clinical Performance**

- The v2.00.00 algorithm demonstrated high diagnostic accuracy in a prospective multicenter external validation (ROMIAE data, ROK 4 hospital EDs; prevalence 22.8%; Patient no. = ECG no. = 1,480). [7]

| AUROC | AUPRC | Cut-off | Sensitivity | Specifity | Positive predictive value | Negative predictive value |
| --- | --- | --- | --- | --- | --- | --- |
| 0.906 (total AMI) | 0.795 | 2.0 | 0.997 | 0.299 | 0.290 | 0.997 |
|  |  | 41.8 | 0.751 | 0.901 | 0.686 | 0.927 |
| 0.979 (STEMI) | 0.910 | 2.0 | 1.000 | 0.298 | 0.141 | 1.000 |
|  |  | 41.8 | 0.954 | 0.901 | 0.525 | 0.994 |
| 0.858 (NSTEMI) | 0.531 | 2.0 | 0.995 | 0.298 | 0.196 | 0.997 |
|  |  | 41.8 | 0.616 | 0.901 | 0.517 | 0.932 |
| 0.879 (Occlusive NSTEMI) | 0.510 | 2.0 | 1.000 | 0.299 | 0.157 | 1.000 |
|  |  | 41.8 | 0.675 | 0.901 | 0.470 | 0.955 |

**Regulatory Approval Status**

- Republic of Korea: Approved July 2025 (v2.00.00). (Previously recognized as an Innovative Medical Device by MFDS in 2023).
- Vietnam: Approved October 2025 (v2.00.00).
- CE-MDR, European Union (EU): Approved March 2026 (v2.00.00)

**References**

1. Song J, Jang JH, Lee BT, et al. Foundation models for ECG: leveraging hybrid self-supervised learning for advanced cardiac diagnostics. arXiv 2024; published online July 10. 2407.07110 (preprint).
2. Johnson AEW, Pollard TJ, Shen L, et al. MIMIC-III, a freely accessible critical care database. Sci Data 2016; 3: 160035.
3. Ribeiro ALP, Ribeiro AH, Paixao GMM, Lima EM, Horta Ribeiro M, Pinto Filho MM, et al. Sami-Trop: 12-lead ECG traces with age and mortality annotations. Zenodo; 2021. DOI:10.5281/zenodo.4905618.
4. Sudlow C, Gallacher J, Allen N, et al. UK Biobank: an open access resource for identifying the causes of a wide range of complex diseases of middle and old age. PLoS Med 2015; 12: e1001779.
5. Ribeiro AH, Paixão GMM, Lima EM, et al. CODE-15%: a large scale annotated dataset of 12-lead ECGs [dataset]. Zenodo 2021. DOI:10.5281/zenodo.4916206.
6. Michal Seják, Jakub Sido, David Žahour. IKEM dataset v1.0.0. Vol 280, Knowledge-Based Systems. [dataset]. Zenodo 2023. DOI:10.5281/zenodo.8393007.
7. Lee MS, Kwon JM, Son JM, Jo YY, Kang S, Lee HS, et al. Early acute myocardial infarction risk stratification in the emergency department: AI-enhanced electrocardiogram and the 10-minute rule [abstract]. J Am Coll Cardiol 2025; 85 (12 suppl): 2749.
8. Lee MS, Shin TG, Lee Y, et al. Artificial intelligence applied to electrocardiogram to rule out acute myocardial infarction: the ROMIAE multicentre study. Eur Heart J 2025; 46: 1917–29.

**Supplemental Tables**

**Supplemental Table 1**

|  | Recruited | **Included** |
| --- | --- | --- |
| **Characteristic** | **N = 17,397***^1^* | **N = 12,630***^1^* |
| Demographics |  |  |
| Age | 60 (47, 74) | 59 (45, 73) |
| Sex (female) | 6,229 (36%) | 4,529 (36%) |
| History, n (%) |  |  |
| History of hypertension | 9,290 (53%) | 6,348 (50%) |
| Dyslipidemia | 7,247 (42%) | 4,692 (37%) |
| Diabetes | 3,013 (17%) | 1,985 (16%) |
| History of CAD | 4,962 (29%) | 3,481 (28%) |
| History of revascularisation | 4,354 (25%) | 2,922 (23%) |
| Vital parameters, median (IQR) |  |  |
| Systolic blood pressure | 139 (125, 155) | 139 (125, 155) |
| Diastolic blood pressure | 82 (73, 91) | 82 (74, 92) |
| Heart rate | 77 (66, 89) | 78 (67, 90) |
| Laboratory findings, median (IQR) |  |  |
| eGFR | 90 (72, 104) | 91 (74, 106) |
| Initial hs-cTnT | 9 (4, 27) | 8 (4, 23) |
| Outcomes, n (%) |  |  |
| Myocardial infarction | 3,807 (22%) | 2,354 (19%) |
| Type 1 myocardial infarction | 3,178 (18%) | 1,900 (15%) |
| Type 2 myocardial infarction | 626 (4%) | 452 (4%) |
| Non-ST-elevation myocardial infarction | 2,648 (15%) | 1,719 (14%) |
| ST-elevation myocardial infarction | 1,159 (7%) | 635 (5%) |
| Occlusion myocardial infarction | 1,167 (7%) | 747 (6%) |
| *^1^*Median (Q1, Q3); n (%) | | |

| **Supplemental Table 2.** | | | | | | | | | | | | | | | | | |  |
| --- | --- | --- | --- | --- | --- | --- | --- | --- | --- | --- | --- | --- | --- | --- | --- | --- | --- | --- |
| **Cohort** | **Age** | | **Sex** | **Discharge**  **diagnosis** | **History of CAD** | **Chest pain onset <3h** | **Baseline hs-cTnT** | **2^nd^**  **hs-cTnT** | **3^rd^**  **hs-cTnT** | **4^th^**  **hs-cTnT** | **5^th^**  **hs-cTnT** | **Physician (%)** | **Basel Score** | **Recalibrated**  **Heart Score** | **AiTiA MI v2.00.00** | **Type1 MI** | **Type 2 MI** | |
| APACE | 48 | male | | Unclear Diagnosis | 0 | 0 | 37.1 | 40.6 | 37.0 |  | 26.1 | 70 | 0 | 4 | 1.3 | 1 | 0 | |
| APACE | 93 | male | | NSTEMI | 1 | 0 | 43.1 | 53.3 | 60.5 | 71.2 |  | 80 | 0 | 7 | 0.9 | 1 | 0 | |
| APACE | 64 | female | | Arrhythmia | 0 | 0 | 94.0 | 101.7 | 109.3 | 115.5 |  | 40 | 0 | 4 | 0.5 | 0 | 1 | |
| APACE | 50 | male | | NSTEMI | 0 | 0 | 265.0 | 275.0 |  |  |  | 90 | 0 | 3 | 1.4 | 1 | 0 | |
| APACE | 86 | female | | NSTEMI | 1 | 0 | 91.0 | 173.0 | 232.0 |  |  | 40 | 1 | 7 | 1.3 | 1 | 0 | |
| APACE | 71 | male | | NSTEMI | 0 | 0 | 21.0 | 23.0 | 23.0 |  |  | 80 | 0 | 6 | 0.3 | 1 | 0 | |
| APACE | 61 | male | | NSTEMI | 0 | 0 | 586.0 |  |  |  |  | 20 | 1 | 6 | 1.7 | 0 | 1 | |
| APACE | 86 | female | | NSTEMI | 1 | 0 | 22.0 | 26.0 |  | 28.0 |  | 90 | 0 | 7 | 1.9 | 1 | 0 | |
| APACE | 81 | male | | NSTEMI | 0 | 1 | 17.0 |  |  | 27.0 | 45.0 | 20 | 0 | 6 | 1.1 | 1 | 0 | |
| APACE | 46 | male | | NSTEMI | 0 | 1 | 21.0 | 34.0 | 30.0 |  |  | 70 | 0 | 6 | 1.7 | 1 | 0 | |
| APACE | 78 | female | | Arrhythmia | 0 | 1 | 13.0 | 18.0 | 22.0 |  | 26.0 | 80 | 0 | 6 | 0.2 | 0 | 1 | |
| APACE | 18 | female | | Arrhythmia | 0 | 1 | 18.0 | 24.0 |  |  |  | 0 | 0 | 2 | 0.1 | 0 | 1 | |
| APACE | 44 | male | | Arrhythmia | 1 | 1 | 35.0 | 49.0 |  | 72.0 |  | 10 | 1 | 6 | 1.5 | 0 | 1 | |
| APACE | 76 | female | | Arrhythmia | 0 | 1 | 17.0 |  | 20.0 |  |  | 10 | 0 | 5 | 0.6 | 0 | 1 | |
| APACE | 26 | male | | Arrhythmia | 1 | 0 | 90.0 | 97.0 | 93.0 |  |  | 40 | 1 | 5 | 0.6 | 0 | 1 | |
| APACE | 46 | female | | NSTEMI | 0 | 0 | 533.0 | 554.0 | 603.0 |  |  | 100 | 0 | 4 | 1.1 | 0 | 1 | |
| APACE | 80 | female | | Arrhythmia | 0 | 1 | 19.0 | 34.0 |  | 43.0 |  | 20 | 0 | 5 | 1.2 | 0 | 1 | |
| APACE | 81 | female | | Unstable Angina | 1 | 1 | 52.0 | 51.0 | 54.0 | 58.0 |  | 70 | 0 | 9 | 0.4 | 1 | 0 | |
| APACE | 53 | male | | NSTEMI | 0 | 0 | 58.0 | 61.0 | 59.0 |  |  | 80 | 0 | 4 | 0.4 | 1 | 0 | |
| APACE | 54 | female | | NSTEMI | 0 | 1 | 43.0 | 55.0 |  |  |  | 90 | 0 | 4 | 1.7 | 0 | 1 | |
| APACE | 77 | female | | Arrhythmia | 0 | 0 | 25.0 | 29.0 |  |  |  | 40 | 0 | 5 | 1.1 | 0 | 1 | |
| APACE | 86 | female | | Unclear Diagnosis | 1 | 0 | 17.0 | 20.0 | 24.0 | 28.0 |  | 0 | 0 | 6 | 0.2 | 0 | 1 | |
| APACE | 37 | female | | Unclear Diagnosis | 0 | 0 | 54.0 | 73.0 |  | 76.0 |  | 0 | 0 | 2 | 0.1 | 0 | 1 | |
| APACE-Light | 44 | male | | STEMI | 0 | 0 | 78 | 68 | 73 | 106 | 103 |  | 1 |  | 0.6 | 0 | 1 | |
| APACE-Light | 80 | male | | NSTEMI | 1 | 0 | 1001 | 1164 | 2238 | 2005 | 1508 |  | 1 |  | 1.8 | 1 | 0 | |
| APACE-Light | 49 | female | | NSTEMI | 0 | 1 | 30 | 101 | 104 | 104 | 58 |  | 0 |  | 0.4 | 0 | 1 | |
| APACE-Light | 49 | female | | NSTEMI | 0 | 0 | 339 | 342 | 309 |  |  |  | 0 |  | 1.2 | 0 | 1 | |
| APACE-Light | 58 | male | | NSTEMI | 0 | 1 | 154 | 214 | 324 | 330 | 316 |  | 0 |  | 1.1 | 0 | 1 | |
| APACE-Light | 82 | male | | COPD | 0 | 0 | 102 | 98 | 91 |  |  |  | 0 |  | 0.4 | 0 | 1 | |
| APACE-Light | 53 | female | | NSTEMI | 0 | 0 | 21 | 36 | 207 | 218 | 200 |  | 0 |  | 1.9 | 1 | 0 | |
| APACE-Light | 51 | male | | Myocardial Infarction | 0 | 1 | 7 | 407 | 322 | 173 | 675 |  |  |  | 0.8 | 1 | 0 | |
| APACE-Light | 62 | female | | NSTEMI | 0 | 1 | 154 | 196 | 237 | 139 |  |  | 0 |  | 0.6 | 0 | 1 | |
| APACE-Light | 80 | female | | NSTEMI | 1 | 1 | 17 | 23 | 33 | 31 | 27 |  | 1 |  | 0.9 | 1 | 0 | |
| APACE-Light | 61 | female | | STEMI | 0 | 1 | 103 | 334 | 761 | 584 | 357 |  | 0 |  | 1.1 | 1 | 0 | |
| APACE-Light | 83 | female | | NSTEMI | 1 | 1 | 35 | 46 | 50 | 62 | 66 |  | 0 |  | 1.6 | 0 | 1 | |
| APACE-Light | 66 | male | | NSTEMI | 1 | 1 | 24 | 42 | 36 | 19 |  |  | 0 |  | 0.5 | 0 | 1 | |
| APACE-Light | 48 | male | | NSTEMI | 1 | 1 | 73 | 73 | 49 | 35 |  |  | 0 |  | 1.4 | 1 | 0 | |
| APACE-Light | 78 | male | | NSTEMI | 1 | 1 | 144 | 133 | 140 | 114 | 126 |  | 1 |  | 0.7 | 1 | 0 | |
| APACE-Light | 61 | female | | NSTEMI | 0 | 1 | 28 | 44 | 53 | 25 |  |  | 0 |  | 1.2 | 0 | 1 | |
| APACE-Light | 52 | female | | NSTEMI | 0 | 1 | 26 | 190 | 241 | 185 |  |  | 1 |  | 0.8 | 0 | 1 | |
| APACE-Light | 47 | female | | NSTEMI | 0 | 1 | 6 | 25 | 144 | 235 | 419 |  | 0 |  | 0.1 | 0 | 1 | |
| APACE-Light | 45 | female | | NSTEMI | 0 | 1 | 31 | 39 | 51 | 47 | 29 |  | 0 |  | 0.3 | 0 | 1 | |
| APACE-Light | 71 | female | | NSTEMI | 0 | 0 | 162 | 163 | 140 | 62 |  |  | 1 |  | 0.4 | 0 | 1 | |
| APACE-Light | 60 | male | | NSTEMI | 0 | 0 | 1278 | 1220 | 1118 | 1394 | 1791 |  | 0 |  | 0.8 | 1 | 0 | |
| APACE-Light | 90 | female | | NSTEMI | 1 | 0 | 146 | 130 |  |  |  |  | 1 |  | 1.8 | 0 | 1 | |
| APACE-Light | 87 | male | | NSTEMI | 1 | 0 | 61 | 61 | 128 | 169 |  |  | 1 |  | 0.2 | 1 | 0 | |
| APACE-Light | 76 | female | | NSTEMI | 1 | 1 | 44 | 90 | 133 | 114 | 116 |  | 0 |  | 1.1 | 1 | 0 | |
| APACE-Light | 72 | female | | NSTEMI | 0 | 1 | 64 | 59 | 55 |  |  |  | 0 |  | 1.8 | 0 | 1 | |
| APACE-Light | 62 | female | | NSTEMI | 0 | 1 | 18 | 52 | 57 |  |  |  | 1 |  | 0.5 | 0 | 1 | |
| APACE-Light | 37 | male | | STEMI | 0 | 1 | 92 | 146 | 165 | 130 |  |  | 0 |  | 0.3 | 1 | 0 | |
| APACE-Light | 64 | male | | NSTEMI | 0 | 1 | 22 | 43 | 61 | 54 | 61 |  | 1 |  | 1.7 | 1 | 0 | |
| APACE-Light | 58 | male | | NSTEMI | 0 | 1 | 16 | 26 | 249 | 482 | 588 |  | 0 |  | 1.1 | 1 | 0 | |
| APACE-Light | 68 | male | | NSTEMI | 1 | 0 | 80 | 118 | 346 | 593 | 353 |  | 0 |  | 1.9 | 1 | 0 | |
| APACE-Light | 86 | male | | NSTEMI | 1 | 0 | 17 | 70 | 171 | 266 | 579 |  | 1 |  | 0.8 | 1 | 0 | |
| APACE-Light | 81 | female | | Myocardial Infarction | 0 | 0 | 18 | 19 | 22 | 24 | 24 |  | 1 |  | 1.7 | 0 | 1 | |
| APACE-Light | 80 | female | | NSTEMI | 1 | 0 | 631 | 633 |  |  |  |  | 1 |  | 1.4 | 0 | 1 | |
| APACE-Light | 80 | female | | NSTEMI | 0 | 0 | 41 | 37 | 32 | 21 | 25 |  | 0 |  | 0.8 | 0 | 1 | |
| CAD: coronary artery disease; Hs-cTnT: High-sensitivity cardiac troponin T; MI: Myocardial infarction; NSTEMI: Non-ST segment elevation myocardial infarction; STEMI: ST segment elevation myocardial infarction; COPD: Chronic obstructive pulmonary disease | | | | | | | | | | | | | | | | | |  |

Supplemental Table 3.

| Cohort | TIMI | n | Median AiTiA MI probability | percentage rule-in | percentage observe | percentage rule-out |
| --- | --- | --- | --- | --- | --- | --- |
| APACE | 0/1 | 207 | 0.8310 | 77.3% | 22.7% | 0.0% |
|  | 2 | 140 | 0.6365 | 66.4% | 32.9% | 0.7% |
|  | 3 | 347 | 0.3550 | 44.1% | 53.3% | 2.6% |
| APACE-Light | 0/1 | 363 | 0.8750 | 80.4% | 19.0% | 0.6% |
|  | 2 | 155 | 0.5720 | 63.9% | 34.2% | 1.9% |
|  | 3 | 391 | 0.4150 | 49.6% | 48.1% | 2.3% |

Supplemental Table 4. TIMI flow distribution among patients with STEMI

| Cohort | TIMI | n | pct |
| --- | --- | --- | --- |
| APACE | 0/1 | 94 | 61.0% |
|  | 2 | 28 | 18.2% |
|  | 3 | 24 | 15.6% |
|  | No PTCA | 8 | 5.2% |
| APACE-Light | 0/1 | 254 | 52.8% |
|  | 2 | 59 | 12.3% |
|  | 3 | 89 | 18.5% |
|  | No PTCA | 70 | 14.5% |
|  | Unknown | 9 | 1.9% |

**Supplemental Figures**

**Supplemental Figure 1.**

**Supplemental Figure 2.**
**Supplemental Figure 3.**

 **Supplemental Figure 4.**

**Supplemental Figure 5.**

**Supplemental Figure 6.**

**Supplemental Figure 7.**

**Supplemental Figure 8.**
**Supplemental Figure 9**

**Supplemental Figure 10.**
